# Sleep Problems and Gambling Disorder: Cross-Sectional Relationships in a Young Cohort

**DOI:** 10.1007/s10899-024-10335-1

**Published:** 2024-07-16

**Authors:** Holly A. Austin, Samuel R. Chamberlain, Jon E. Grant, David S. Baldwin

**Affiliations:** 1 Clinical and Experimental Sciences, Faculty of Medicine, University of Southampton, Southampton, UK; 2 Southern Health NHS Foundation Trust, Southampton, UK; 3 Department of Psychiatry & Behavioral Neuroscience, University of Chicago, Chicago, USA; 4 University Department of Psychiatry and Mental Health, University of Cape Town, Cape Town, South Africa

**Keywords:** Gambling disorder, Sleep, Insomnia, Depression, Anxiety

## Abstract

**Aims:**

To investigate the potential association between gambling disorder and symptoms of sleep problems (including insomnia and excessive daytime sleepiness). It was hypothesised that, compared to controls, individuals with gambling disorder would have significantly greater disturbance of sleep, as indicated by increased scores in: (1) sleep items on the Hamilton Anxiety Rating Scale (HAM-A) and Hamilton Rating Scale for Depression (HAM-D); (2) total score on the HAM-A and HAM-D; and (3) the Epworth Sleepiness Scale (ESS).

**Methods:**

Secondary analysis of previously published data from 152 young adults, aged 18–29 years. Individuals were stratified into three groups: controls, those at risk of gambling disorder, and those with gambling disorder. One-way ANOVAs with post-hoc tests were conducted to determine whether groups differed significantly in sleep item scores and total scores of the HAM-A and HAM-D, and the ESS.

**Results:**

HAM-D scale insomnia item scores were significantly higher in the disorder group, when compared to controls, this being particularly marked for middle and late insomnia. The HAM-A item score indicated significantly worse sleep quality in the disorder group, compared to at risk and control groups. Total HAM-A and HAM-D scores were significantly higher in the disorder group, but ESS scores did not differ significantly.

**Conclusion:**

Measures of disruptions in sleep were significantly higher in gambling disorder than controls. Anxiety and depressive symptom severity was also significantly higher in the gambling disorder group. Further research could have implications for identification and treatment of sleep disorders and psychiatric comorbidities in gambling disorder.

## Background

Insomnia is a common sleep problem: chronic insomnia affects around 10% of the adult population (Morin et al., 2006). It is also an important and independent risk factor for mental health problems, including depression, anxiety and suicide, and for physical health problems including cardiovascular disease (Riemann et al., 2022). Sleep disturbance is ubiquitous in presentations relating to poor mental health and is likely to be a contributory causal factor in the occurrence of many mental health problems (Freeman et al., 2020). As well as being an independent risk factor for developing mental health problems, insomnia is a common current co-morbidity for individuals suffering with mental health problems, including depression (Geoffroy et al., 2018).

Excessive daytime sleepiness is defined as “an inability to stay awake or alert during major waking episodes resulting in periods of irrepressible need for sleep or unintended lapses into drowsiness or sleep.” (Sateia, 2014). It is common in the general population, with a reported prevalence of 23.34% in American adults and is associated with a higher chance of having a DSM-5 mental disorder (OR = 4.25; 95% CI 3.53–5.10), (Kolla et al., 2020). Excessive daytime sleepiness can have behavioural causes such as chronic sleep restriction due to poor sleep habits; physical causes such as obstructive sleep apnoea, central disorders of hypersomnolence, including narcolepsy and idiopathic hypersomnia; and psychological causes such as depression (Pérez-Carbonell et al., 2022).

Sleep problems are also common in certain addictions. A review concluded that insomnia can be a significant risk factor for onset of substance use disorders (including alcohol and opiates), and sleep impairments can increase risk of relapse (Reid-Varley et al., 2020).

Gambling Disorder is a psychiatric disorder characterised by a persistent, recurrent pattern of gambling that is associated with substantial distress or impairment, over a 12 month period (American Psychiatric Association 2013). In a meta-analysis of recent available literature, the population prevalence of gambling disorder was estimated to be 0.6-2% (95% confidence interval) (Gabellini et al., 2023). There is increasing recognition of Gambling Disorder as a serious public health concern, with specialist clinics being opened within the National Health Service in the UK (Metcalfe, 2023). A recent paper argued for gambling disorder to be conceptualised as “gambling dual disorder” due to its frequent co-occurrence with other mental health disorders including substance use disorder, attention deficit/hyperactivity disorder (ADHD) and mood disorders. It also highlighted the co-occurrence of other addictive behaviours such as compulsive spending, but did not consider sleep problems (Szerman et al., 2023).

Previous investigation of the relationship between sleep disturbances and gambling behaviour has been limited. Parhami et al. investigated a community sample of 96 gamblers, who were recruited via online and newspaper adverts, but excluded if they were receiving or seeking treatment for their gambling difficulties – therefore the examined sample was non-treatment seeking (Parhami et al., 2012). They found significantly worse sleep quality (indicated by higher scores in the Pittsburgh Sleep Quality Index (Buysse et al., 1989) and Epworth Sleepiness Scales (Johns, 1991) among problem and pathological gamblers as compared to controls: this was the first study to use objective sleep measures in this population. In a nationally representative survey, 85 participants with problem gambling behaviour and 31 with pathological gambling were compared to 3305 controls: pathological gamblers were at increased risk of difficulties in initiating sleep (2.3 times), and maintaining sleep (4.6 times), and early morning awakening (4.0 times) (Parhami et al., 2013). Finally, a study involving over 3500 treatment-seeking gamblers found that those with insomnia were more likely to report both suicidal ideation and familial-suicidal ideation; however this was assessed with a dichotomous “yes/no” question about the presence of insomnia (Wong et al., 2014).

Our study aimed to extend current knowledge, by exploring potential associations between gambling disorder and sleep problems.

## Methods

### Data Collection

The dataset derives from earlier work on impulsivity and sleepiness and uses a subset of previously published data (Grant & Chamberlain, 2018). Inclusion criteria were: (1) male or female between the ages of 18–29; (2) gambled at least 5 times in the past year; and (3) ability to understand the procedures and provide written consent. Exclusion criteria were inability to understand the procedures and provide written consent.

Problem gambling behaviours were quantified using the Structured Clinical Interview for Gambling Disorder adapted from (Grant et al., 2004) to consider DSM-5 gambling disorder. The number of criteria met allowed stratification of individuals into the following groups: Controls (SCIPG = 0), At Risk gambling (SCIPG = 1–3), and Gambling Disorder (SCIPG ≥ 4).

The original sample comprised 579 individuals recruited from two metropolitan areas and incorporated a variety of measures including cognitive assessments and impulsivity. In this investigation, we particularly wanted to examine the relationship between level of gambling disorder, Epworth Sleepiness Scale score (Johns, 1991) and specific questions relating to insomnia from the Hamilton Anxiety Rating Scale (HAM-A) (Hamilton, 1959) and Hamilton Rating Scale for Depression (HAM-D) (Hamilton, 1960). Therefore, we included only individuals with complete data for these 4 measures; resulting in 152 individuals from the Chicago area (See Table 1 - Demographics).

**Table 1:** Demographics of the young adult sample grouped based on current gambling severity

	*Controls* (*n* = 63)	*At Risk* (*n* = 49)	*Gambling Disorder* (*n* = 40)
Mean Age (SD)	23.97 (2.98)	24.94 (2.98)	25.69 (2.97)
Male, n (%)	28 (44%)	27 (55%)	28 (70%)
Female, n (%)	35 (56%)	22 (45%)	12 (30%)
**Ethnicity**,** n (%)**			
Caucasian	35 (56%)	21 (43%)	7 (18%)
African-American	21 (33%)	18 (37%)	25 (63%)
Latino/Hispanic	3 (5%)	8 (16%)	1 (3%)
Asian	1 (2%)	1 (2%)	1 (3%)
Native American	1 (2%)	0	1 (3%)
Middle Eastern	0	0	0
Blank/not specified	0	1 (2%)	1 (3%)
Mixed	2 (3%)	0	3 (8%)
**Education**			
Less than high school	1 (2%)	0	1 (3%)
High school graduate	5 (8%)	4 (8%)	8 (20%)
Some college	21 (33%)	27 (55%)	19 (48%)
Graduated college	20 (32%)	15 (31%)	11 (28%)
More than college	16 (25%)	3 (6%)	1 (3%)
**Employment**,** n (%)**			
Full time	18 (29%)	20 (41%)	13 (33%)
Part Time	11 (17%)	13 (27%)	11 (28%)
Student	10 (16%)	1 (2%)	6 (15%)
Unemployed	11 (17%)	9 (18%)	10 (25%)
Retired	0	0	0
Student and Employed	11 (17%)	6 (12%)	0

The Epworth Sleepiness Scale (Johns, 1991) is a patient-rated scale for assessing the likeliness of falling asleep in 8 situations, from sitting and reading to being in a car. Each question is rated 0–3 (would never doze – high chance of dozing), for a total score between 0 and 24. Scores > 10 are considered to reflect excessive daytime sleepiness.

The HAM-A is a clinician-rated scale with 14 items to assess anxiety; each item is scored on a scale of 0 (not present) to 4 (severe), for a total score range of 0–56, where a score of 18–24 typically indicates mild to moderate severity and 25–30 moderate to severe (Hamilton, 1959). There is a specific item on the HAM-A that assesses global disruptions in sleep, rating “difficulty in falling asleep, broken sleep, unsatisfying sleep and fatigue on waking, dreams, nightmares, night terrors”, from 0 (not present) to 4 (very severe).

The HAM-D is a clinician-rated scale to measure depression, with 17 items rated either from 0 to 4 or 0–2 depending on the symptom assessed; for a total score out of 61 (Hamilton, 1960). Mild depression typically corresponds to scores of 8–16, moderate depression to scores of 17–23, and severe depression to scores of *≥* 24 (Zimmerman et al., 2013). There are 3 specific items for rating sleep, these are “insomnia – initial” (difficulty falling asleep), insomnia – middle (restlessness and disturbed sleep during the night, waking during the night), and insomnia – delayed (waking in early hours of the morning and an inability to fall asleep again). These 3 questions are each rated as 0 (absent), 1 (occasional) or 2 (frequent).

### Study Hypotheses

The study hypotheses were determined a priori as follows:

1) There will be a significant difference in Epworth Sleepiness Scale scores between the three groups, with highest scores in Gambling Disorder, followed by At Risk gamblers, versus Controls.

2) There will be a significant difference in HAM-A and HAM-D total scores between the three groups, with highest scores in Gambling Disorder, followed by At Risk gamblers, versus Controls.

3) There will be a significant difference in sleep questions on the HAM-A and HAM-D across the three groups, with highest scores in Gambling Disorder, followed by At Risk gamblers, versus Controls.

### Data Analysis

To examine potential differences between the groups on the demographic and clinical measures of interest, analysis of variance (ANOVA) tests were conducted for continuous variables, and chi-square tests for categorical variables. *Post-hoc* Tukey Honestly Significant Difference (HSD) tests were performed to compare the significance of differences between all the possible pairs of means. Statistical significance was defined as *p* < 0.05.

Statistical analysis was undertaken using SPSS Statistics for Windows v 28 (IBM Corp., 2021).

## Results

### Demographics

The overall sample of 152 participants had a mean (standard deviation) age of 24.7 (2.9) years and 54.6% (*n* = 83) were male. The Control, At Risk and Gambling Disorder groups are considered separately in Table 1.

Analyses showed that marital status and sexual orientation did not differ significantly between the three groups. Age was significantly different between the groups (*p* = 0.013), with post hoc Tukey’s HSD test showing a significant difference between the Controls and the Gambling Disorder patients (Gambling Disorder participants are on average older), but not a significant difference between the At Risk group and the Controls, or the At Risk and the Disorder group. Gender was significantly different between groups (*p* = 0.04), with more males than females in the Disorder group and employment status was also significantly different (*p* = 0.0008) with a higher proportion of unemployed people in the Disorder group and less students in the At Risk group. Finally ethnicity was also significantly different (*p* = 0.004) with more African-Americans in the disorder group, and more Latinos in the At Risk group, compared to Controls.

### Results for ESS, HAM-A and HAM-D Scales

Focusing on the Epworth Sleepiness Scale, there was no significant difference in total Epworth Score between the groups (*p* = 0.106). There was also no difference in the typical sleep hours between the groups as rated on the ESS (*p* = 0.054). Data on typical sleep hours was present for 61/63 participants in the Control group, 47/49 in the At Risk group and 39/40 in the Disorder group. For all the other domains measured, all data were present for all participants (*n* = 152). The clinical cut off for excessive daytime sleepiness is generally considered as a score of 11 or greater (Trimmel et al., 2018). In the groups, 16 of the 63 Controls, 16 of the 49 At Risk group, and 15 of the 40 Disorder group scored > 10, and there was no significant difference between groups (*p* = 0.411).

Considering the HAM-A; the total score was significantly different between the 3 groups (*p* < 0.001). *Post-hoc* tests showed the Disorder group was significantly different to the Controls and the At Risk group. The Disorder group had a mean score of 9.85 on the HAM-A, compared to 6.08 and 3.95 in the At Risk and Control Groups, respectively. The insomnia item on the HAM-A was also significantly different (ANOVA; *p* < 0.02), with *post-hoc* tests showing significant differences between the Disorder compared to the Control and At Risk groups.

For the HAM-D; there was a significant difference in total score between the groups on ANOVA (*p* < 0.001); *post-hoc* tests showed significant differences between the Disorder group compared to the Control and At Risk groups. The Disorder group had a mean total score of 12.03, compared to 6.61 and 4.17 in the Control and At Risk groups, respectively.

There was a significant difference on the Early Insomnia item score of the HAM-D between the 3 groups (*P* < 0.043); *post hoc* analysis showed the significant difference was between the Disorder and the Control group, but not the At Risk group.

For the middle and late insomnia items, there were significant differences between groups on ANOVA of 0.004 and 0.002 respectively. *Post hoc* tests showed these differences were between the Disorder group compared to the Control and At Risk groups.

The results are summarised in Table 2. Values that do not share a letter are significantly different, for each measurement (*p* < 0.05), as per Tukey’s HSD post-hoc tests. This allows comparison of each group to the other two.

**Table 2:** Clinical characteristics of young adults grouped by gambling severity

Measurement	Mean Value (SD) Controls (*n*=63)	At Risk (*n* = 49)	Disorder (*n* = 40)	ANOVA F	*P* (significance)
ESS Score	7.05 (4.89)	8.55 (4.39)	8.90 (5.07)	2.28	0.106
ESS typical sleep hours	6.98 (1.11)	6.97 (1.40)	6.39 (1.37)	2.98	0.054
HAM-A total score	3.95 (3.97)^a^	6.08 (6.17)^a^	9.85 (7.47)^b^	12.72	< 0.001
HAM-A insomnia question item 4	0.83 (0.87)^a^	1.04 (1.06)^a^	1.58 (1.17)^b^	6.71	0.02
HAM-D total score	4.17 (4.00)^a^	6.61 (5.38)^a^	12.03 (7.71)^b^	24.07	< 0.001
HAM-D4 early insomnia	0.60 (0.71)^a^	0.69 (0.74)^a, b^	0.98 (0.77)^b^	3.22	0.043
HAM-D5 middle insomnia	0.37 (0.60)^a^	0.43 (0.68)^a^	0.83 (0.87)^b^	5.62	0.004
HAM-D6 late insomnia	0.44 (0.64)^a^	0.49 (0.71)^a^	0.95 (0.86)^b^	6.72	0.002

*Abbreviations* ESS – Epworth Sleepiness Scale, HAM-A Hamilton Anxiety Rating Scale, HAM-D Hamilton Rating Scale for Depression, ANOVA – Analysis of Variance, SD – Standard Deviation

## Discussion

We investigated two types of sleep symptoms in a stratified gambling population: excessive daytime sleepiness, measured using the Epworth Sleepiness Scale; and insomnia, measured by specific item questions on the Hamilton Anxiety Rating Scale (HAM-A) and Hamilton Rating Scale for Depression (HAM-D). The study groups of interest were: people with Gambling Disorder, people with At Risk Gambling (i.e. endorsing some but not sufficient criteria for a gambling disorder diagnosis), and Controls (people who gamble recreationally but meet no diagnostic criteria for gambling disorder).

We hypothesised that there would be a significant difference in the Epworth Sleepiness Scale scores between the three groups, with Gambling Disorder being associated with highest scores, and the At Risk group occupying an intermediate position versus Controls. The findings indicate that although scores were numerically higher in the Disorder group, the difference between groups overall was not statistically significant. Furthermore, none of the three groups had a mean score indicating clinical significance on the Epworth Scale, which is generally considered to be a score of > 10. Therefore hypothesis #1 was incorrect.

The findings that HAM-D scale insomnia item scores were significantly higher in the Disorder group, when compared to Controls, particularly for middle and late insomnia, is similar to the pattern of insomnia previously described in Gambling Disorder (Parhami et al., 2013). A possible source of difference between the two scales is that the Epworth is self-administered whereas the HAM-A and HAM-D are administered by staff.

Early morning wakening is also typically associated with more severe depression (although some studies dispute this (Bjorøy et al., 2020)), which can be screened for using the HAM-D. The Gambling Disorder group had a mean total score of 12.03 on HAM-D - this would correspond to mild depression (score 10–13). The other groups did not fall within this range. This comorbid depression may partially explain the apparent association between gambling disorder and early waking.

Similarly, the HAM-A item score (global sleep disruption question) indicated significantly worse sleep quality in the Disorder group, compared to the At Risk and Control groups. HAM-A scores were significantly higher in individuals suffering from gambling disorder (*p* < 0.01), with the Gambling Disorder patient group having a mean score of 12.03 - corresponding to mild anxiety (score 8–14) in the Gambling Disorder group compared to below cut-off range reflecting no or minimal signs of anxiety in the other groups.

Therefore, our second study hypothesis, that there will be a significant difference in HAM-A and HAM-D total scores between the three groups, with highest scores in Gambling Disorder, followed by At Risk gamblers, versus Controls was supported. However *post-hoc* tests showed the significant difference for total scores was between the Disorder group and the other 2 groups; the Controls and At Risk groups were not significantly different to each other.

Our third hypothesis, that there will be a significant difference in sleep questions on the HAM-A and HAM-D across the three groups was supported, with highest scores in Gambling Disorder, followed by At Risk gamblers, versus Controls. However *post-hoc* tests showed the significant difference the individual sleep items was between the Disorder group and the other 2 groups; the Controls and At Risk were not significantly different to each other, with the exception of the early insomnia question on the HAM-D. For this item, the 3 groups were each significantly different from each other.

These findings indicate that whilst there were significant differences between the Control and the Disorder groups, the At Risk group was not as distinct as we anticipated. In almost every domain studied it was significantly different from the Gambling Disorder group, but not the Controls.

Overall, the relationship between sleep problems and gambling is poorly understood. An investigation in a small ecological sample of regular online poker players (*n* = 23) found that hands of poker played whilst sleep deprived were more likely to have a negative financial outcome, and participants were more likely to report a loss of control when gambling whilst sleep-deprived. In addition, hands played within two hours of bedtime were not impacting sleep quality, and participants reported falling asleep faster after playing a hand close to bedtime, the authors suggesting that participants found the poker to be a relaxing and pleasurable activity, resulting in this shorter sleep onset latency (Hamel et al., 2021). Future work could examine motivation to gamble and sleep variables in larger samples and in other types of gambling.

Given the apparent increase in sleep problems associated with gambling addiction, specific questions regarding insomnia may be needed within clinical settings, as asking a question that focusses on initial insomnia, such as “do you have difficulty falling asleep?” may not be as sensitive in this group, nor pick up sleep difficulties later in the night. Considering an individual patient’s day and night-time routine and their association between poor sleep and gambling could be useful. Treatments for insomnia such as Cognitive Behavioural Therapy for Insomnia (CBT-I) are widely available, but have not been evaluated specifically for gambling disorder patients. Another clinical consideration is that this study underlines the importance of screening for other mental health disorders, as there were higher rates of anxiety and depression in the Gambling Disorder group than in Controls.

## Limitations

One limitation relates to the employed scales. Some previous studies (Perlis et al., 2006; Serretti et al., 2005) have used the HAM-D insomnia questions as a marker for insomnia: however, the HAM-A and HAM-D scales were designed for assessing severity of anxiety and depression, not sleep disturbance. Future work should consider using specific sleep scales, such as the Pittsburgh Sleep Quality Index (Buysse et al., 1989) or Insomnia Severity Index (Morin, 1993), to ascertain their validity in gambling disorder patients, and deploy them to explore sleep issues.

Another limitation is the sample size; we could not specifically examine the effects of known confounders such as smoking and comorbidities such as ADHD and caffeine consumption which may have influenced sleep patterns. Larger sample sizes from more diverse populations both demographically and geographically may help advance this field and allow the examination for confounders. Additionally, this sample was composed of young adults, not seeking treatment. A previous study identified demographic and clinical differences in treatment-seekers compared to non-treatment seekers, in alcohol misuse disorder subjects, and the same may apply to gambling disorder patients (Ray et al., 2017). Further research could investigate sleep patterns in older adults and in those who have sought treatment.

Lastly it should be noted that the Control group in this study comprised people who gamble at least occasionally but do not meet any diagnostic criteria for gambling disorder. If recreational gambling is itself linked to sleep problems, this could have contributed to some of the non-significant findings between controls and the other study groups. Findings may of course differ if comparing people with Gambling Disorder, or At Risk gambling, to controls who do not gamble at all.

## Conclusion

Global disruptions in sleep, and late- and middle-insomnia, were significantly greater in individuals with Gambling Disorder when compared to Controls. Symptoms of anxiety and depression were also significantly more severe in the Gambling Disorder group. The At Risk group was not significantly distinct to the Control group in scores on most of the scales studies, with the exception of the “early insomnia” item on the HAM-D.

Further research could have implications for the identification and treatment of sleep disorders and psychiatric comorbidities in gambling disorder.

## Data Availability

The data that support the findings of this study are not openly available due to reasons of sensitivity and are available from the authors upon reasonable request.
